# Crystal structure of melaminium cyano­acetate monohydrate

**DOI:** 10.1107/S2056989020012335

**Published:** 2020-09-11

**Authors:** Bhawani Sigdel Regmi, Allen Apblett, Douglas Powell

**Affiliations:** a Department of Chemistry and Biochemistry, University of North Georgia, Oakwood, Georgia, USA; b Oklahoma State University, Stillwater, Oklahoma, USA; c University of Oklahoma, Norman, Oklahoma, USA

**Keywords:** crystal structure, melaminium cation, cyano­acetate anion, hydrogen bonding

## Abstract

The asymmetric unit of the title compound, 2,4,6-tri­amino-1,3,5-triazin-1-ium cyano­acetate monohydrate, C_3_H_7_N_6_^+^·NCCH_2_COO^−^·H_2_O, consists of a melaminium cation, a cyano­acetate anion and a water mol­ecule, which are connected to each other *via* N—H⋯O and O—H⋯O hydrogen bonds, generating an eight-membered ring. In the crystal, the melaminium cations are connected by two pairs of N—H⋯N hydrogen bonds, forming tapes along [110]. These tapes develop a three-dimensional network through N—H⋯O, O—H⋯O, N—H⋯N and C—H⋯O hydrogen bonds between the cations, anions and water mol­ecules.

## Abstract 2

The crystal of the melaminium salt C_3_H_7_N_6_^+^·NCCH_2_COO^−^·H_2_O was produced by mixing melamine with cyano­acetic acid in aqueous solution. The melaminium cations are inter­connected by N—H⋯N hydrogen bonds, forming tapes. These tapes of melaminium cations develop a three-dimensional network through multiple donor–acceptor hydrogen-bonding inter­actions between the cyano­acetate anions and water mol­ecules.

## Chemical context   

Melamine (systematic name: 2,4,6-tri­amino-1,3,5-triazine), a trimer of cyanamide, has many industrial applications. The cross-linked resins of melamine with formaldehyde have applications in adhesive coatings, laminations and flame retardants (Billmeyer, 1984 ▸). In the past, various organic melamine salts were tested as potential melamine substitutes for melamine urea formaldehyde resins (Weinstabl *et al.*, 2001 ▸). In general, protonation of melamine with organic and inorganic acids has been found to yield compounds with extensive hydrogen-bonding networks involving both N—H⋯O and O—H⋯O hydrogen bonds. This paper is a part of our investigation of the chemistry of cyano­acetate with nitro­gen-based cations and their potential application as flame retardants since cyano­acetic acid is an analogue to polyacrylo­nitrile. It is well known that polyacrylo­nitrile is used in industry to manufacture carbon fibers because of its ability to produce carbon char (Bacon & Hoses, 1986 ▸). Cyano­acetic acid has a nitrile group and also can act as acid source, both of which could enhance the flame-retarding properties.
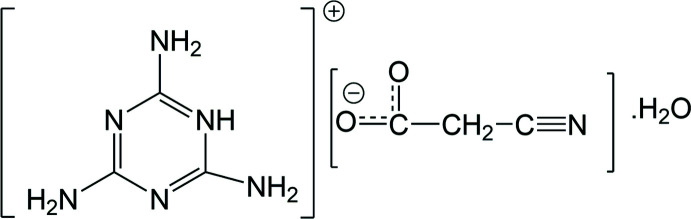


## Structural commentary   

**Figure 1:**
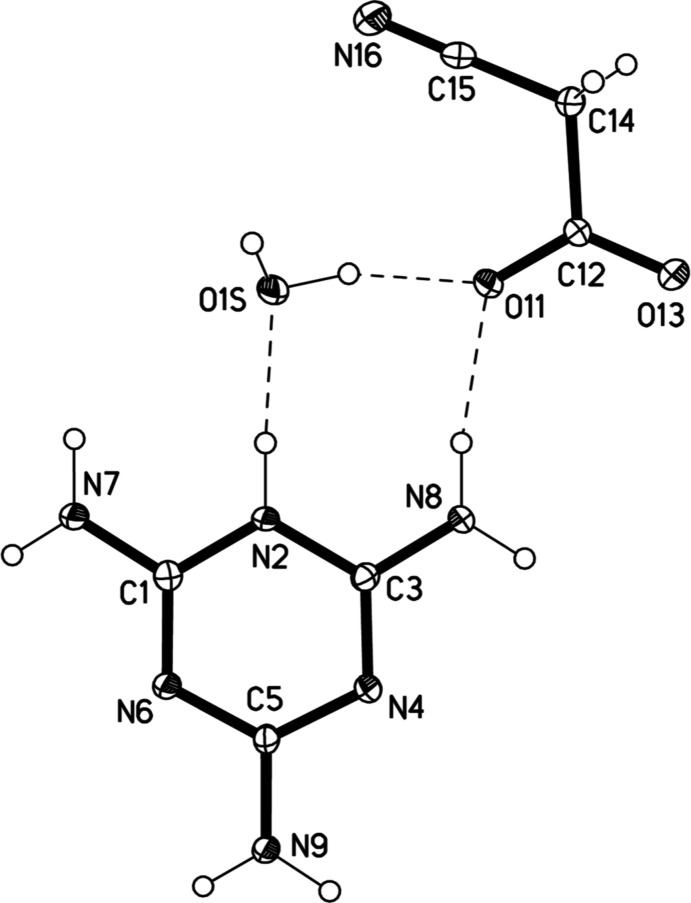
Mol­ecular structure of the title compound, showing 50% probability displacement ellipsoids and the atom-numbering scheme. Hydrogen atoms are shown as spheres of arbitrary radius and hydrogen bonds as dashed lines.

The asymmetric unit of the title compound consists of a melaminium cation, a cyano­acetate anion and a water mol­ecule, which are connected to each other *via* N—H⋯O and O—H⋯O hydrogen bonds, generating an eight-membered ring (Fig. 1 ▸). The six-membered ring of the melaminium cation shows significant distortion from a hexa­gonal shape. The bond distances [C—N = 1.322 (2)–1.368 (2) Å] and the angles [C—N—C = 115.76 (15)–119.08 (14)° and N—C—N = 121.44 (15)–125.42 (15)°] fall within similar ranges to those reported for similar singly protonated melaminium salts of simple alkyl mono- and di­carb­oxy­lic acids, namely, melaminium acetate acetic acid solvate (Perpétuo & Janczak, 2002 ▸), melaminium maleate (Janczak & Perpétuo, 2004 ▸), melaminium formate (Perpétuo *et al.*, 2005 ▸), melaminium tartarate (Su *et al.*, 2009 ▸), bis­(melaminium) succinate (Froschauer & Weil, 2012*a* ▸) and melaminium hydrogen malonate (Froschauer & Weil, 2012*b* ▸). On the other hand, the angles in the six-membered ring of unprotonated melamine (Adam *et al.*, 2010 ▸) are in the range 124.86 (17) to 125.51 (17)°.

In the anion, both O atoms of the carboxyl­ate group are involved in hydrogen bonds to amino groups of adjacent melaminium ions. The nitrile group has a bond length of 1.145 (2) Å that is typical of a nitrile (Kanters *et al.*, 1978 ▸). The angle at the nitrile carbon, N≡C—C, is 179.30 (19)° which is close to the theoretical value of 180°. The O atom of the water mol­ecule acts as a lone-pair donor to the protonated nitro­gen of the melaminium ion that is present in the same eight-membered ring. The presence of the water mol­ecule in the structure of melaminium cyano­acetate can be expected to contribute to fire retardancy as its release and evaporation will provide cooling.

## Supra­molecular features   

**Figure 2:**
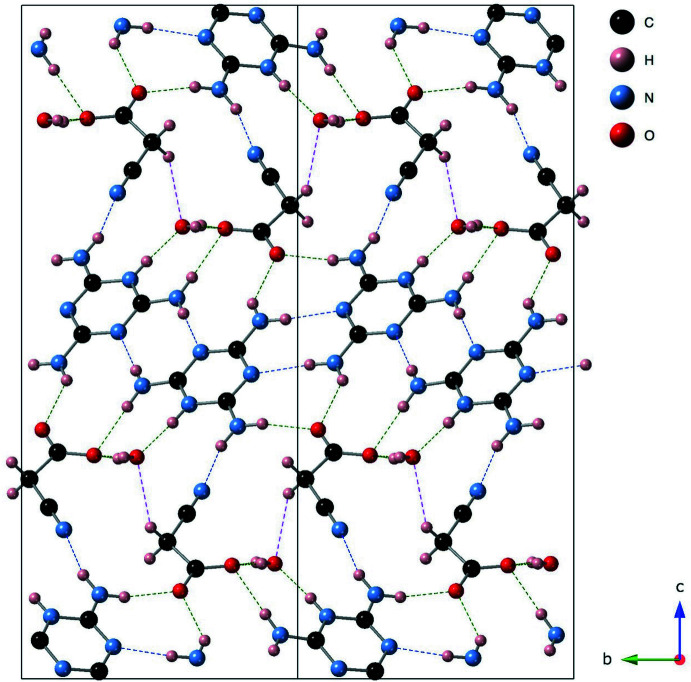
A packing diagram of the title compound, viewed down the *a* axis, showing the O—H⋯O and N—H⋯O hydrogen bonds (green dashed lines), the N—H⋯N hydrogen bonds (blue dashed lines) and the C—H⋯O hydrogen bonds (magenta dashed lines).

The melaminium cation in the crystal is involved in altogether nine hydrogen bonds: for each melaminium cation, seven of them are of the hydrogen-bond donor type while the remaining two are of the acceptor type (Table 1 ▸). Neighbouring cations are connected by two pairs of N—H⋯N hydrogen bonds (N8—H8*B*⋯N4^iii^ and N9—H9*B*⋯N6^iv^; symmetry codes as in Table 1 ▸) to form a tape-like structure propagating along [110] and running between the cyano­acetate anions. Three N—H⋯O hydrogen bonds (N7—H7*A*⋯O13^i^, N8—H8*A*⋯O11 and N9—H9*A*⋯O13^iii^; Table 1 ▸) link the cation with three different cyano­acetate anions. Furthermore, the cation is also connected with a water mol­ecule *via* an N—H⋯O hydrogen bond (N2—H2⋯O1*S*) between the protonated imine and the water O atom. Finally, the cation is linked with the nitrile group of the anion *via* an N—H⋯N hydrogen bond (N7—H7*B*⋯N16^ii^; Table 1 ▸). There also exist O—H⋯O (O1*S*—H1*SA*⋯O11 and O1*S*—H1*SB*⋯O11^vi^) hydrogen bonds between the water mol­ecule and the anion. In addition, a C—H⋯O hydrogen bond between the methyl­ene H and water O atoms is observed as the C—H group is activated because of the electron-withdrawing cyano group adjacent to it. Altogether, these hydrogen bonds existing between the cations, anions and water mol­ecules generate a three-dimensional network (Fig. 2 ▸).

## Database survey   

A search of the Cambridge Structural Database (Version 5.40, update of May 2020; Groom *et al.*, 2016 ▸) for 2,4,6-tri­amino-1,3,5-triazin-1-ium showed more than 30 records; however, for 2,4,6-tri­amino-1,3,5-triazin-1-ium forming only single protonated salts with purely organic aliphatic carb­oxy­lic acids the search gave the following crystal structures: melamine with maleic acid (refcode ARUDAS; Janczak & Perpétuo, 2004 ▸), with formic acid (FONMEB; Perpétuo *et al.*, 2005 ▸), with acetic acid (EFAZOA; Perpétuo & Janczak, 2002 ▸), with malonic acid (HOWRIV01; Froschauer & Weil, 2012*b* ▸), with succinic acid (LEGZEE; Froschauer & Weil, 2012*a* ▸), with nitrilo­tri­acetic acid (MIHYAF; Hoxha *et al.*, 2013 ▸) and with tartaric acid (VORSUR; Su *et al.*, 2009 ▸). A search for organic co-crystals/salts of cyano­acetic acid gave one structure, 4,4′-bi­pyridine bis­(cyano­acetic acid) (Song *et al.*, 2008 ▸). For metal complexes with cyano­acetic acid or cyano­acetate, 24 structures were reported, such as silver cyano­acetate (Edwards *et al.*, 1997 ▸) and cadmium cyano­acetate (Post & Trotter, 1974 ▸). In these metal salts, the metal is coordinated by the acetate group as well as the cyano group.

## Synthesis and crystallization   

A solution of cyano­acetic acid (1.7g, 20 mmol) in 100 ml of deionized water was added to a solution of melamine (2.5 g, 20 mmol) in 100 ml of deionized water. The reaction mixture was heated to 353 K for 3 h. The resulting clear solution was cooled to room temperature and then was allowed to slowly evaporate. Single crystals of the title compound formed after several days.

## Refinement   

Crystal data, data collection and structure refinement details are summarized in Table 2 ▸. C-bound H atoms were initially determined by geometry (C—H = 0.99 Å) and were refined using a riding model, with *U*_iso_(H) = 1.2*U*_eq_(C). H atoms bonded to N and O were located in a difference map, and their positions were refined freely, with *U*_iso_(H) = 1.2*U*_eq_(N or O).

## Supplementary Material

Crystal structure: contains datablock(s) I, General. DOI: 10.1107/S2056989020012335/is5552sup1.cif

Structure factors: contains datablock(s) I. DOI: 10.1107/S2056989020012335/is5552Isup2.hkl

Supporting information file. DOI: 10.1107/S2056989020012335/is5552Isup3.cml

CCDC reference: 2030784

Additional supporting information:  crystallographic information; 3D view; checkCIF report

## Figures and Tables

**Table 1:** Hydrogen-bond geometry (Å, °)

*D*—H⋯*A*	*D*—H	H⋯*A*	*D*⋯*A*	*D*—H⋯*A*
N2—H2⋯O1*S*	0.90 (2)	1.81 (2)	2.7067 (19)	176.4 (19)
N7—H7*A*⋯O13^i^	0.89 (2)	2.00 (2)	2.881 (2)	168 (2)
N7—H7*B*⋯N16^ii^	0.92 (2)	2.13 (2)	3.001 (2)	155.6 (18)
N8—H8*A*⋯O11	0.91 (2)	2.01 (2)	2.891 (2)	164.6 (19)
N8—H8*B*⋯N4^iii^	0.88 (2)	2.07 (2)	2.952 (2)	176 (2)
N9—H9*A*⋯O13^iii^	0.90 (2)	2.08 (2)	2.792 (2)	135.7 (19)
N9—H9*B*⋯N6^iv^	0.90 (2)	2.08 (2)	2.980 (2)	174 (2)
C14—H14*B*⋯O1*S* ^v^	0.99	2.46	3.233 (2)	134
O1*S*—H1*SA*⋯O11	0.93 (2)	1.78 (2)	2.6860 (19)	163.3 (19)
O1*S*—H1*SB*⋯O11^vi^	0.87 (2)	1.97 (2)	2.8351 (19)	178 (2)

Symmetry codes: (i) 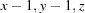; (ii) 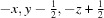; (iii) 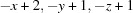; (iv) 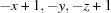; (v) 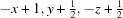; (vi) 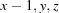.

**Table 2:** Experimental details

Crystal data	
Chemical formula	C_3_H_7_N_6_^+^·C_3_H_2_NO_2_^−^·H_2_O
*M*_r_	229.22
Crystal system, space group	Monoclinic, *P*2_1_/*c*
Temperature (K)	100
*a*, *b*, *c* (Å)	4.6928 (6), 9.3881 (13), 22.918 (3)
β (°)	91.646 (3)
*V* (Å^3^)	1009.3 (2)
*Z*	4
Radiation type	Mo *K*α
μ (mm^−1^)	0.12
Crystal size (mm)	0.44 × 0.17 × 0.04

Data collection	
Diffractometer	Bruker *APEX* CCD
Absorption correction	Multi-scan (*SADABS*; Bruker, 2007 ▸)
*T*_min_, *T*_max_	0.948, 0.995
No. of measured, independent and observed [*I* > 2σ(*I*)] reflections	12615, 2517, 1856
*R*_int_	0.058
(sin θ/λ)_max_ (Å^−1^)	0.668

Refinement	
*R*[*F* ^2^ > 2σ(*F* ^2^)], *wR*(*F* ^2^), *S*	0.047, 0.126, 1.00
No. of reflections	2517
No. of parameters	172
H-atom treatment	H atoms treated by a mixture of independent and constrained refinement
Δρ_max_, Δρ_min_ (e Å^−3^)	0.28, −0.29

Computer programs: *SMART* and *SAINT* (Bruker, 2007 ▸), *SHELXT* (Sheldrick, 2015*a* ▸), *SHELXL2018/3* (Sheldrick, 2015*b* ▸), *SHELXTL* (Sheldrick, 2008 ▸) and *CrystalMaker* (Palmer, 2014 ▸).

## supplementary crystallographic information

### Crystal data

**Table:**

C_3_H_7_N_6_^+^·C_3_H_2_NO_2_^−^·H_2_O	*F*(000) = 480
*M**_r_* = 229.22	*D*_x_ = 1.509 Mg m^−^^3^
Monoclinic, *P*2_1_/*c*	Mo *K*α radiation, λ = 0.71073 Å
*a* = 4.6928 (6) Å	Cell parameters from 3720 reflections
*b* = 9.3881 (13) Å	θ = 2.3–28.1°
*c* = 22.918 (3) Å	µ = 0.12 mm^−^^1^
β = 91.646 (3)°	*T* = 100 K
*V* = 1009.3 (2) Å^3^	Needle, colourless
*Z* = 4	0.44 × 0.17 × 0.04 mm

### Data collection

**Table:**

Bruker APEX CCD diffractometer	1856 reflections with *I* > 2σ(*I*)
φ and ω scans	*R*_int_ = 0.058
Absorption correction: multi-scan (*SADABS*; Bruker, 2007)	θ_max_ = 28.4°, θ_min_ = 1.8°
*T*_min_ = 0.948, *T*_max_ = 0.995	*h* = −5→6
12615 measured reflections	*k* = −12→12
2517 independent reflections	*l* = −30→30

### Refinement

**Table:**

Refinement on *F*^2^	Primary atom site location: structure-invariant direct methods
Least-squares matrix: full	Secondary atom site location: difference Fourier map
*R*[*F*^2^ > 2σ(*F*^2^)] = 0.047	Hydrogen site location: mixed
*wR*(*F*^2^) = 0.126	H atoms treated by a mixture of independent and constrained refinement
*S* = 1.00	*w* = 1/[σ^2^(*F*_o_^2^) + (0.064*P*)^2^ + 0.320*P*] where *P* = (*F*_o_^2^ + 2*F*_c_^2^)/3
2517 reflections	(Δ/σ)_max_ = 0.001
172 parameters	Δρ_max_ = 0.28 e Å^−^^3^
0 restraints	Δρ_min_ = −0.29 e Å^−^^3^

### Special details

**Table:**

Geometry. All esds (except the esd in the dihedral angle between two l.s. planes) are estimated using the full covariance matrix. The cell esds are taken into account individually in the estimation of esds in distances, angles and torsion angles; correlations between esds in cell parameters are only used when they are defined by crystal symmetry. An approximate (isotropic) treatment of cell esds is used for estimating esds involving l.s. planes.

### Fractional atomic coordinates and isotropic or equivalent isotropic displacement parameters (Å^2^)

**Table:**

	*x*	*y*	*z*	*U*_iso_*/*U*_eq_	
C1	0.3297 (4)	0.25701 (18)	0.41214 (7)	0.0117 (4)	
N2	0.4321 (3)	0.39286 (15)	0.40750 (6)	0.0121 (3)	
H2	0.359 (4)	0.453 (2)	0.3804 (9)	0.014*	
C3	0.6562 (4)	0.43434 (17)	0.44283 (7)	0.0111 (4)	
N4	0.7614 (3)	0.35064 (14)	0.48477 (6)	0.0126 (3)	
C5	0.6396 (4)	0.21954 (17)	0.48861 (8)	0.0125 (4)	
N6	0.4316 (3)	0.16816 (15)	0.45231 (6)	0.0135 (3)	
N7	0.1226 (3)	0.21696 (16)	0.37493 (7)	0.0151 (3)	
H7A	0.059 (4)	0.128 (2)	0.3786 (9)	0.018*	
H7B	0.055 (4)	0.277 (2)	0.3458 (9)	0.018*	
N8	0.7672 (3)	0.56251 (15)	0.43490 (7)	0.0137 (3)	
H8A	0.711 (4)	0.617 (2)	0.4041 (9)	0.016*	
H8B	0.914 (5)	0.586 (2)	0.4577 (9)	0.016*	
N9	0.7332 (4)	0.13535 (16)	0.53094 (7)	0.0202 (4)	
H9A	0.870 (5)	0.163 (2)	0.5569 (10)	0.024*	
H9B	0.673 (5)	0.044 (3)	0.5337 (9)	0.024*	
O11	0.6998 (3)	0.73778 (13)	0.33172 (5)	0.0157 (3)	
C12	0.7511 (4)	0.87001 (18)	0.33632 (8)	0.0129 (4)	
O13	0.9314 (3)	0.92554 (14)	0.36958 (6)	0.0224 (3)	
C14	0.5789 (4)	0.97232 (18)	0.29669 (8)	0.0163 (4)	
H14A	0.465226	1.035861	0.321467	0.020*	
H14B	0.713248	1.032642	0.275142	0.020*	
C15	0.3874 (4)	0.90159 (18)	0.25472 (8)	0.0156 (4)	
N16	0.2366 (4)	0.84731 (17)	0.22144 (7)	0.0237 (4)	
O1S	0.2181 (3)	0.58309 (13)	0.32906 (6)	0.0163 (3)	
H1SA	0.363 (5)	0.650 (2)	0.3269 (9)	0.020*	
H1SB	0.059 (5)	0.630 (2)	0.3289 (9)	0.020*	

### Atomic displacement parameters (Å^2^)

**Table:**

	*U*^11^	*U*^22^	*U*^33^	*U*^12^	*U*^13^	*U*^23^
C1	0.0117 (8)	0.0117 (8)	0.0118 (8)	−0.0003 (6)	0.0012 (6)	−0.0011 (6)
N2	0.0141 (8)	0.0088 (7)	0.0131 (7)	−0.0006 (6)	−0.0040 (6)	0.0023 (6)
C3	0.0116 (9)	0.0105 (8)	0.0110 (8)	0.0009 (6)	−0.0005 (6)	−0.0011 (6)
N4	0.0140 (8)	0.0095 (7)	0.0141 (7)	−0.0014 (6)	−0.0023 (6)	0.0011 (5)
C5	0.0123 (9)	0.0104 (7)	0.0148 (9)	−0.0017 (6)	−0.0024 (7)	0.0005 (6)
N6	0.0161 (8)	0.0099 (7)	0.0144 (8)	−0.0012 (6)	−0.0048 (6)	0.0016 (6)
N7	0.0184 (8)	0.0109 (7)	0.0155 (8)	−0.0025 (6)	−0.0063 (6)	0.0025 (6)
N8	0.0148 (8)	0.0108 (7)	0.0152 (8)	−0.0039 (6)	−0.0036 (6)	0.0036 (6)
N9	0.0257 (9)	0.0117 (7)	0.0222 (9)	−0.0067 (7)	−0.0141 (7)	0.0064 (6)
O11	0.0150 (7)	0.0108 (6)	0.0208 (7)	−0.0009 (5)	−0.0040 (5)	0.0034 (5)
C12	0.0135 (9)	0.0115 (8)	0.0136 (9)	−0.0024 (7)	−0.0013 (7)	0.0022 (6)
O13	0.0269 (8)	0.0163 (6)	0.0231 (7)	−0.0048 (6)	−0.0135 (6)	0.0034 (5)
C14	0.0197 (10)	0.0113 (8)	0.0174 (9)	−0.0010 (7)	−0.0069 (7)	−0.0001 (7)
C15	0.0164 (9)	0.0128 (8)	0.0174 (9)	0.0025 (7)	−0.0027 (7)	0.0037 (7)
N16	0.0273 (10)	0.0198 (8)	0.0235 (9)	−0.0010 (7)	−0.0104 (7)	0.0012 (7)
O1S	0.0129 (7)	0.0126 (6)	0.0232 (7)	−0.0003 (5)	−0.0031 (5)	0.0052 (5)

### Geometric parameters (Å, º)

**Table:**

C1—N6	1.322 (2)	N8—H8B	0.88 (2)
C1—N7	1.329 (2)	N9—H9A	0.90 (2)
C1—N2	1.368 (2)	N9—H9B	0.90 (2)
N2—C3	1.365 (2)	O11—C12	1.268 (2)
N2—H2	0.90 (2)	C12—O13	1.238 (2)
C3—N4	1.326 (2)	C12—C14	1.536 (2)
C3—N8	1.326 (2)	C14—C15	1.458 (2)
N4—C5	1.361 (2)	C14—H14A	0.9900
C5—N9	1.317 (2)	C14—H14B	0.9900
C5—N6	1.353 (2)	C15—N16	1.145 (2)
N7—H7A	0.89 (2)	O1S—H1SA	0.93 (2)
N7—H7B	0.92 (2)	O1S—H1SB	0.87 (2)
N8—H8A	0.91 (2)		
			
N6—C1—N7	120.78 (16)	C3—N8—H8A	121.0 (13)
N6—C1—N2	121.44 (15)	C3—N8—H8B	117.0 (14)
N7—C1—N2	117.77 (15)	H8A—N8—H8B	121.5 (19)
C3—N2—C1	119.08 (14)	C5—N9—H9A	122.0 (14)
C3—N2—H2	120.1 (13)	C5—N9—H9B	121.5 (14)
C1—N2—H2	120.8 (13)	H9A—N9—H9B	116 (2)
N4—C3—N8	119.86 (16)	O13—C12—O11	126.04 (16)
N4—C3—N2	121.68 (15)	O13—C12—C14	116.09 (15)
N8—C3—N2	118.45 (15)	O11—C12—C14	117.87 (15)
C3—N4—C5	115.76 (15)	C15—C14—C12	114.17 (14)
N9—C5—N6	117.29 (15)	C15—C14—H14A	108.7
N9—C5—N4	117.30 (16)	C12—C14—H14A	108.7
N6—C5—N4	125.42 (15)	C15—C14—H14B	108.7
C1—N6—C5	116.32 (15)	C12—C14—H14B	108.7
C1—N7—H7A	116.4 (13)	H14A—C14—H14B	107.6
C1—N7—H7B	121.4 (13)	N16—C15—C14	179.30 (19)
H7A—N7—H7B	122.1 (19)	H1SA—O1S—H1SB	106.4 (19)
			
N6—C1—N2—C3	4.1 (2)	C3—N4—C5—N6	2.5 (3)
N7—C1—N2—C3	−176.16 (16)	N7—C1—N6—C5	−179.16 (16)
C1—N2—C3—N4	−5.8 (2)	N2—C1—N6—C5	0.6 (2)
C1—N2—C3—N8	174.98 (16)	N9—C5—N6—C1	176.40 (17)
N8—C3—N4—C5	−178.19 (16)	N4—C5—N6—C1	−4.1 (3)
N2—C3—N4—C5	2.6 (2)	O13—C12—C14—C15	174.83 (17)
C3—N4—C5—N9	−177.98 (17)	O11—C12—C14—C15	−4.3 (3)

### Hydrogen-bond geometry (Å, º)

**Table:**

*D*—H···*A*	*D*—H	H···*A*	*D*···*A*	*D*—H···*A*
N2—H2···O1*S*	0.90 (2)	1.81 (2)	2.7067 (19)	176.4 (19)
N7—H7*A*···O13^i^	0.89 (2)	2.00 (2)	2.881 (2)	168 (2)
N7—H7*B*···N16^ii^	0.92 (2)	2.13 (2)	3.001 (2)	155.6 (18)
N8—H8*A*···O11	0.91 (2)	2.01 (2)	2.891 (2)	164.6 (19)
N8—H8*B*···N4^iii^	0.88 (2)	2.07 (2)	2.952 (2)	176 (2)
N9—H9*A*···O13^iii^	0.90 (2)	2.08 (2)	2.792 (2)	135.7 (19)
N9—H9*B*···N6^iv^	0.90 (2)	2.08 (2)	2.980 (2)	174 (2)
C14—H14*B*···O1*S*^v^	0.99	2.46	3.233 (2)	134
O1*S*—H1*SA*···O11	0.93 (2)	1.78 (2)	2.6860 (19)	163.3 (19)
O1*S*—H1*SB*···O11^vi^	0.87 (2)	1.97 (2)	2.8351 (19)	178 (2)

Symmetry codes: (i) *x*−1, *y*−1, *z*; (ii) −*x*, *y*−1/2, −*z*+1/2; (iii) −*x*+2, −*y*+1, −*z*+1; (iv) −*x*+1, −*y*, −*z*+1; (v) −*x*+1, *y*+1/2, −*z*+1/2; (vi) *x*−1, *y*, *z*.
